# Supplementary material for: Associations of cardiovascular health and social determinants of health with the risks of all-cause and cause-specific mortality
Source: PLoS One. 2025 Nov 24;20(11):e0337286. doi: 10.1371/journal.pone.0337286 (PMC12643303; doi:10.1371/journal.pone.0337286)
Supplement: S1 Fig — (DOCX) [file pone.0337286.s001.docx]

**S1 Fig. Flowchart of the study.**

**
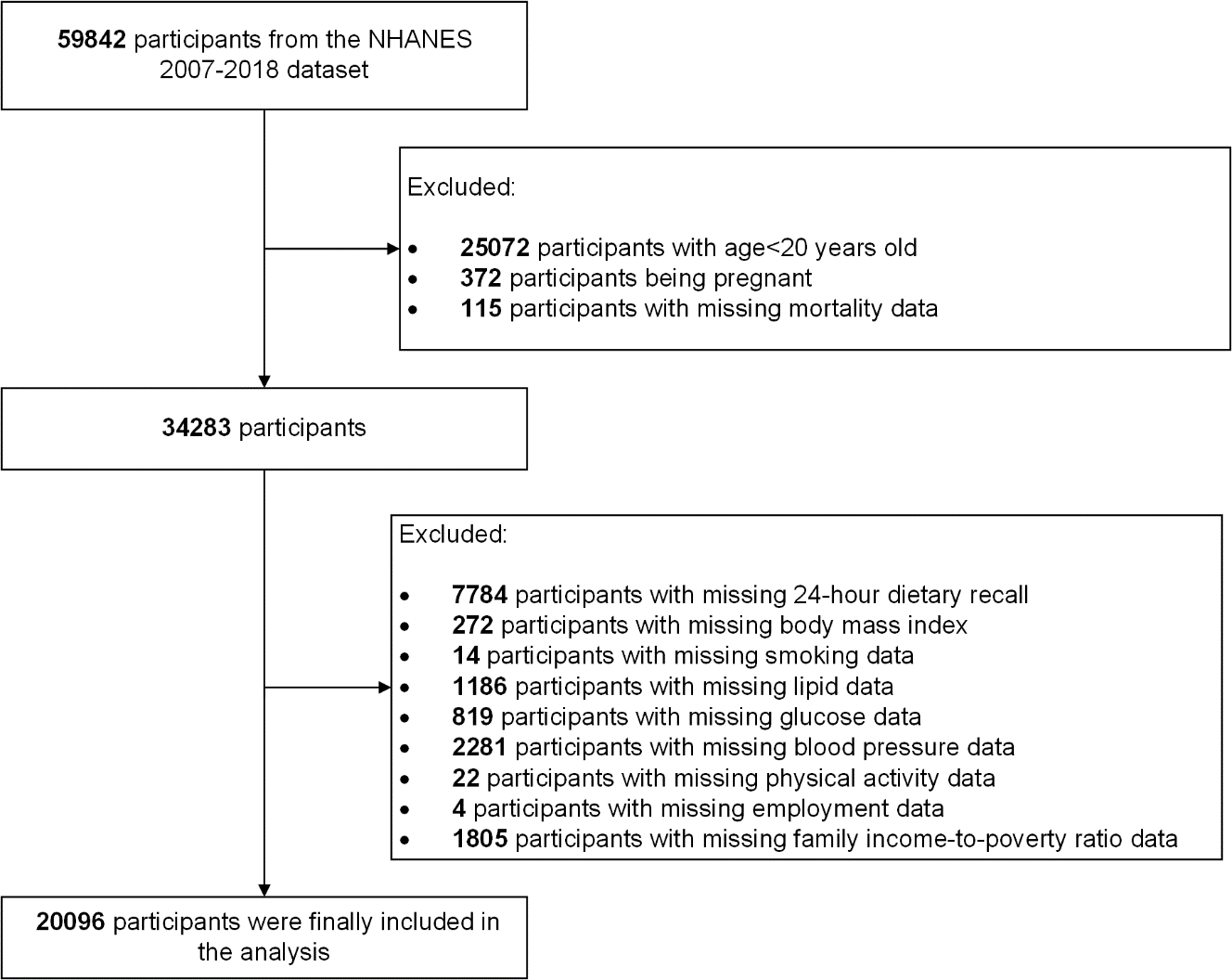
**

Abbreviation: NHANES: the National Health and Nutrition Examination Survey.
