# Supplementary material for: Associations of cardiovascular health and social determinants of health with the risks of all-cause and cause-specific mortality
Source: PLoS One. 2025 Nov 24;20(11):e0337286. doi: 10.1371/journal.pone.0337286 (PMC12643303; doi:10.1371/journal.pone.0337286)
Supplement: S1 Table — (DOCX) [file pone.0337286.s002.docx]

**S1 Table. Methods for evaluating each single cardiovascular health metric.**

| **CVH metric** | **Method of measurement** | **Quantification of CVH metric** | |
| --- | --- | --- | --- |
| **HEI-2015 score*** |  | **Points** | **HEI-2015 diet score** |
|  | Using dietary data collected from 2 nonconsecutive 24-hour recalls. The mean values of each dietary component were used in this study | 100 | 95th percentile (top/ideal diet) |
|  |  | 80 | 75th–94th percentile |
|  |  | 50 | 50th–74th percentile |
|  |  | 25 | 25th–49th percentile |
|  |  | 0 | 1st–24th percentile (bottom/ least ideal quartile) |
| **Physical activity score** |  | **Points** | **Minutes of moderate- (or greater) intensity physical activity per week** |
|  | Self-reported minutes of moderate or vigorous physical activity per week | 100 | ≥150 minutes |
|  |  | 90 | 120-149 minutes |
|  |  | 80 | 90-119 minutes |
|  |  | 60 | 60-89 minutes |
|  |  | 40 | 30-59 minutes |
|  |  | 20 | 1-29 minutes |
|  |  | 0 | 0 minutes |
| **Tobacco/nicotine exposure score** |  | **Points** | **Combustible tobacco use or secondhand smoke exposure** |
|  | Self-reported tobacco use or secondhand smoke exposure | 100 | Never smoker |
|  |  | 75 | Former smoker, quit ≥ 5 years |
|  |  | 50 | Former smoker, quit 1–<5 years |
|  |  | 25 | Former smoker, quit <1 years |
|  |  | 0 | Current smoker |
|  |  | Subtract 20 points (unless score is 0) for living with active indoor smoker in home | |
| **Sleep health score** |  | **Points** | **Sleep hours** |
|  | Self-reported average  hours of sleep per night | 100 | 7-9 hours |
|  |  | 90 | 9-<10 hours |
|  |  | 70 | 6–<7 hours |
|  |  | 40 | 5–<6 or ≥10 hours |
|  |  | 20 | 4–<5 hours |
|  |  | 0 | < 4 hours |
| **Body mass index score** |  | **Points** | **BMI** |
|  | BMI was calculated as weight in kilograms divided by standing height in meters squared. Weight and standing height were measured in mobile examination centers with standard protocols. | 100 | <25 kg/m2 |
|  |  | 70 | 25.0-29.9 kg/m^2^ |
|  |  | 30 | 30.0-34.9 kg/m^2^ |
|  |  | 15 | 35.0-39.9 kg/m^2^ |
|  |  | 0 | ≥ 40.0 kg/m^2^ |
|  |  |  | |
| **Blood lipid score** |  | **Points** | **Non-HDL cholesterol** |
|  | Non-HDL cholesterol was calculated by total cholesterol minus HDL cholesterol. Serum cholesterol was measured enzymatically | 100 | <130 mg/dL |
|  |  | 60 | 130-159 mg/dL |
|  |  | 40 | 160-189 mg/dL |
|  |  | 20 | 190-219 mg/dL |
|  |  | 0 | ≥ 220 mg/dL |
|  |  | If drug-treated level, subtract 20 points | |
| **Glucose score** |  | **Points** | **FPG or HbA1c** |
|  | HbA1c was measured by high-performance liquid chromatography methods. FPG was measured by standard methods. | 100 | No history of diabetes and  FPG <100 mg/dL (or HbA1c <5.7 %) |
|  |  | 60 | No diabetes and FPG 100–125 mg/dL (or HbA1c 5.7–6.4%) |
|  |  | 40 | Diabetes with HbA1c <7.0 % |
|  |  | 30 | Diabetes with HbA1c 7.0–7.9 % |
|  |  | 20 | Diabetes with HbA1c 8.0–8.9 % |
|  |  | 10 | Diabetes with Hb A1c 9.0–9.9 % |
|  |  | 0 | Diabetes with HbA1c ≥10.0 % |
| **Blood pressure score** |  | **Points** | **Systolic and diastolic BPs** |
|  | The average of all available BP measurements was used to calculate systolic and diastolic BP. BPs were measured in mobile examination centers with standard protocols. | 100 | <120/<80 mm Hg |
|  |  | 75 | 120-129/<80 mm Hg |
|  |  | 50 | 130-139 or 80-89 mm Hg |
|  |  | 25 | 140-159 or 90-99 mm Hg |
|  |  | 0 | ≥160 or ≥100 mm Hg |
|  |  | Subtract 20 points (unless score is 0) if treated level | |

* The healthy eating index-2015 (HEI-2015) includes 9 adequacy components (total fruit, whole fruit, total vegetables, greens and beans, whole grains, dairy, total protein foods, seafood and plant protein, and fatty acids) and 4 moderation components (refined grains, sodium, percentage of energy from added sugars, and percentage of energy from saturated fatty acids). The total HEI-2015 score ranges from 0 to 100 points. A higher score reflects healthier eating quality.

Abbreviations: CVH: cardiovascular health; HEI-2015: healthy eating index-2015; BMI: body mass index; HDL: high-density lipoprotein; BP: blood pressure; FPG: fasting plasma glucose; HbA1c: hemoglobin A1.
