# Supplementary material for: Associations of cardiovascular health and social determinants of health with the risks of all-cause and cause-specific mortality
Source: PLoS One. 2025 Nov 24;20(11):e0337286. doi: 10.1371/journal.pone.0337286 (PMC12643303; doi:10.1371/journal.pone.0337286)
Supplement: S3 Table — (DOCX) [file pone.0337286.s004.docx]

**S3 Table.** **Baseline characteristics of participants included or excluded from the current analysis.**

| **Characteristics** | **Excluded** | **Included** | **P value** |
| --- | --- | --- | --- |
| Weighted N (weighted %) | 212,446,305  (59.18) | 146,521,183 (40.82) |  |
| No. of participants in sample | 39746 | 20096 |  |
| Age, years (SE) | 37.77 (0.29) | 47.83 (0.28) | <0.001 |
| Women, n (weighted %) | 20151 (51.15) | 10254 (51.38) | 0.406 |
| Race and ethnicity, n (weighted %) |  |  | 0.177 |
| Mexican | 6510 (10.55) | 2863 (7.78) |  |
| White | 13094 (61.22) | 9098 (70.18) |  |
| Black | 9301 (12.07) | 4000 (9.84) |  |
| Other | 10847 (16.17) | 4135 (12.20) |  |
| Medical history, n (weighted %) |  |  |  |
| CVD history | 2613 (6.47) | 2144 (8.21) | <0.001 |
| Cancer history | 2268 (5.62) | 1972 (10.40) | <0.001 |
| SDoH, n (weighted %) |  |  |  |
| Unemployed | 6725 (18.44) | 7714 (31.26) | <0.001 |
| Family income-to-poverty ratio<300% | 30545 (66.48) | 12535 (48.78) | <0.001 |
| Marginal or lower food security | 15967 (32.39) | 6232 (23.13) | <0.001 |
| Not owning a home | 19631 (40.61) | 7435 (30.70) | <0.001 |
| Less than high school | 29405 (60.57) | 4363 (13.85) | <0.001 |
| No regular health care access | 4883 (13.45) | 3608 (16.67) | <0.001 |
| No private health insurance | 23019 (46.59) | 9283 (35.71) | <0.001 |
| Not married or living with a partner | 31520 (70.62) | 7938 (35.29) | <0.001 |
| AHA LE8 score, mean (SE) |  |  |  |
| Total CVH score | 61.18 (0.34) | 61.03 (0.32) | 0.626 |
| HEI-2015 diet score | 22.34 (0.25) | 39.01 (0.24) | <0.001 |
| Physical activity score | 47.90 (0.82) | 46.36 (0.83) | 0.025 |
| Tobacco/nicotine exposure score | 70.55 (0.45) | 72.13 (0.55) | 0.009 |
| Sleep health score | 49.24 (0.49) | 70.04 (1.02) | <0.001 |
| Body mass index score | 76.91 (0.37) | 60.32 (0.42) | <0.001 |
| Blood lipid score | 81.95 (0.30) | 63.43 (0.38) | <0.001 |
| Blood glucose score | 89.32 (0.33) | 79.61 (0.31) | <0.001 |
| Blood pressure score | 67.16 (0.18) | 57.35 (0.27) | <0.001 |

Data are survey-weighted mean (SE) or N (weight percentage %).

Abbreviations: SE: standard error; CVD: cardiovascular diseases; SDoH: social determinants of health; CVH: cardiovascular health; AHA: American Heart Association; LE8: Life’s Essential 8; HEI-2015: healthy eating index-2015.
