# Supplementary material for: Associations of cardiovascular health and social determinants of health with the risks of all-cause and cause-specific mortality
Source: PLoS One. 2025 Nov 24;20(11):e0337286. doi: 10.1371/journal.pone.0337286 (PMC12643303; doi:10.1371/journal.pone.0337286)
Supplement: S4 Table — (DOCX) [file pone.0337286.s005.docx]

S4 Table. Association between social determinants of health and cardiovascular health.

| **Unfavorable SDoH** | **Unadjusted** | |  | **Adjusted** | |  |
| --- | --- | --- | --- | --- | --- | --- |
|  | **β (95% CI)** | **P value** |  | **β (95% CI)** | **P value** |  |
| CVH score | -1.88 (-1.98- -1.78) | <0.001 |  | -1.91 (-2.00- -1.81) | <0.001 |  |

Model was adjusted for age, sex, race/ethnicity, cardiovascular disease history, and cancer history. Abbreviations: SDoH: social determinants of health; CVH: cardiovascular health; CI: confidence interval.
