# Supplementary material for: Associations of cardiovascular health and social determinants of health with the risks of all-cause and cause-specific mortality
Source: PLoS One. 2025 Nov 24;20(11):e0337286. doi: 10.1371/journal.pone.0337286 (PMC12643303; doi:10.1371/journal.pone.0337286)
Supplement: S2 Fig — (DOCX) [file pone.0337286.s006.docx]

**S2 Fig. Mediation effects of cardiovascular health on the association of social determinants of health with mortality.**

**
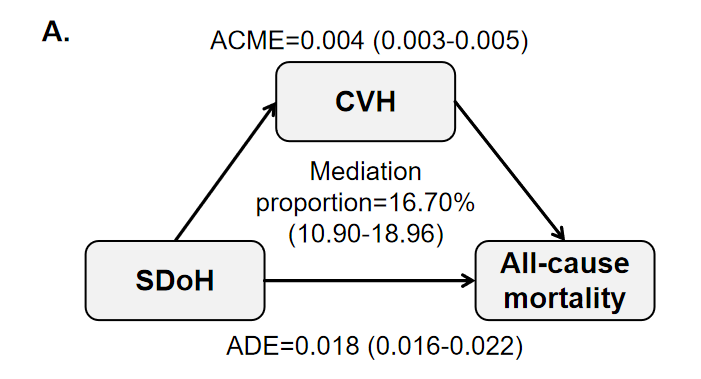
**

**
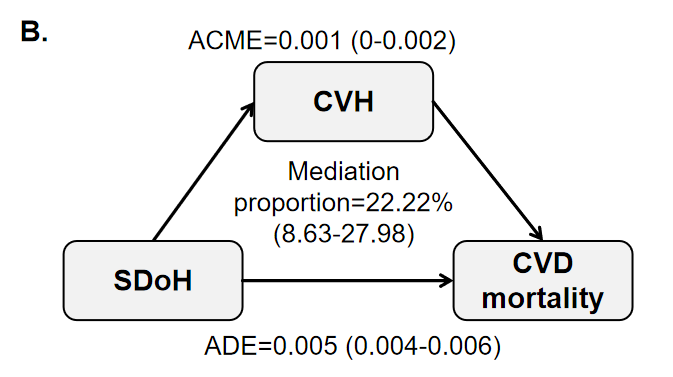
**

**
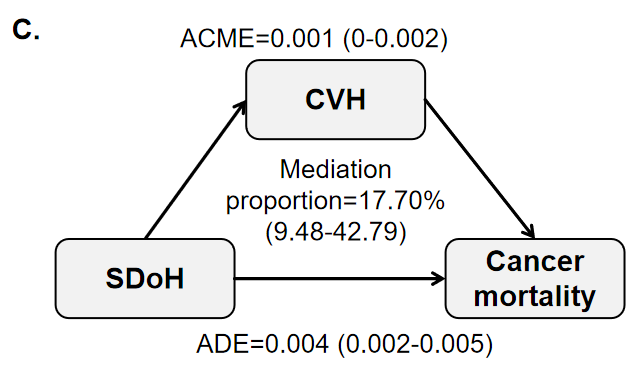
**

Mediation effects of CVH on the association between SDoH and all-cause mortality (A); CVD mortality (B); cancer mortality (C).

Abbreviations: SDoH: social determinants of health; CVH: cardiovascular health; CVD: cardiovascular diseases; ACME: average causal mediation effect; ADE: average direct effect.
