# Supplementary material for: Associations of cardiovascular health and social determinants of health with the risks of all-cause and cause-specific mortality
Source: PLoS One. 2025 Nov 24;20(11):e0337286. doi: 10.1371/journal.pone.0337286 (PMC12643303; doi:10.1371/journal.pone.0337286)
Supplement: S5 Table — (DOCX) [file pone.0337286.s007.docx]

**S5 Table. Predictive value of models without and with the social determinants of health.**

| **Comparison** | **Differences of C-index**  **(95% CI)** | **P_c-index_** | **NRI**  **(95% CI)** | **P_NRI_** | **IDI (95% CI)** | **P_IDI_** |
| --- | --- | --- | --- | --- | --- | --- |
| **All-cause mortality** |  |  |  |  |  |  |
| Adjusted for SDoH+CVH vs. Adjusted for CVH | 0.012  (0.010-0.020) | <0.001 | 0.256  (0.214-0.272) | <0.001 | 0.008  (0.003-0.013) | <0.001 |
| **CVD mortality** |  |  |  |  |  |  |
| Adjusted for SDoH+CVH vs. Adjusted for CVH | 0.007  (0.001-0.008) | <0.001 | 0.168  (0.120-0.210) | <0.001 | 0.006  (0.002-0.012) | <0.001 |
| **Cancer mortality** |  |  |  |  |  |  |
| Adjusted for SDoH+CVH vs. Adjusted for CVH | 0.006  (0.001-0.007) | <0.001 | 0.083  (0.021-0.133) | 0.020 | 0.003  (0.001-0.006) | <0.001 |

All models included age, sex, race/ethnicity, cardiovascular disease history, and cancer history.

Abbreviations: SDoH: social determinants of health; CI: confidence interval; CVH: cardiovascular health; NRI: net reclassification index; IDI: integrated discrimination improvement.
