# Supplementary material for: Associations of cardiovascular health and social determinants of health with the risks of all-cause and cause-specific mortality
Source: PLoS One. 2025 Nov 24;20(11):e0337286. doi: 10.1371/journal.pone.0337286 (PMC12643303; doi:10.1371/journal.pone.0337286)
Supplement: S3 Fig — (DOCX) [file pone.0337286.s008.docx]

**S3 Fig. Association of cardiovascular health with all-cause and cause-specific mortality stratified by levels of social determinants of health.**


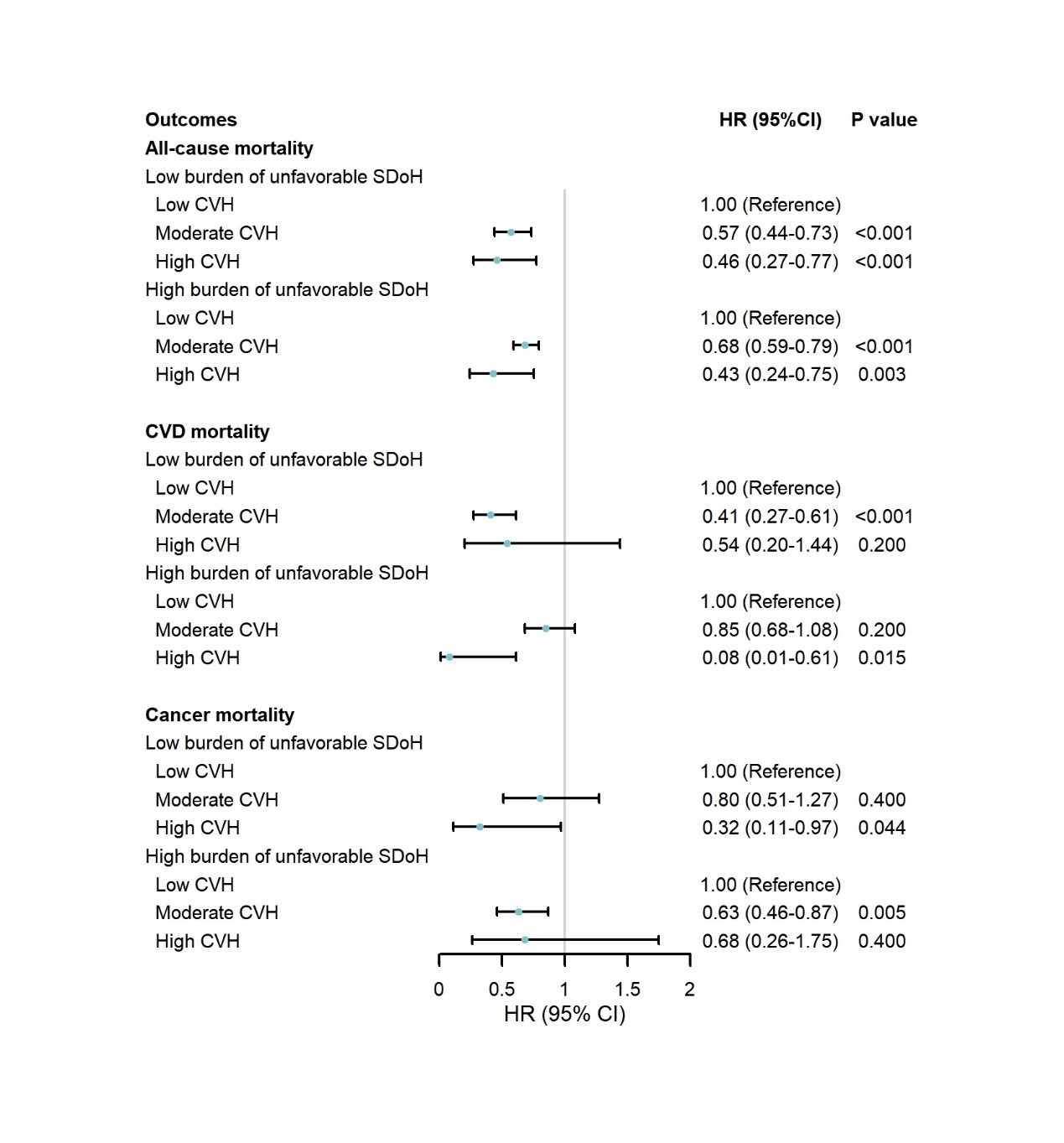


Multivariable models were adjusted for age, sex, race/ethnicity, cardiovascular disease history, and cancer history.

Abbreviations: SDoH: social determinants of health; CVH: cardiovascular health; HR: hazard ratio; CI: confidence interval; CVD: cardiovascular diseases.
