# Supplementary material for: Associations of cardiovascular health and social determinants of health with the risks of all-cause and cause-specific mortality
Source: PLoS One. 2025 Nov 24;20(11):e0337286. doi: 10.1371/journal.pone.0337286 (PMC12643303; doi:10.1371/journal.pone.0337286)
Supplement: S6 Table — (DOCX) [file pone.0337286.s009.docx]

**S6 Table. Characteristics of participants by combined categories of social determinants of health and cardiovascular health.**

| **Characteristics** | **Low burden of unfavorable SDoH** | | | **High burden of unfavorable SDoH** | | | **P value** |
| --- | --- | --- | --- | --- | --- | --- | --- |
|  | **High**  **CVH** | **Moderate CVH** | **Low**  **CVH** | **High**  **CVH** | **Moderate CVH** | **Low**  **CVH** |  |
| Weighted N (weighted %) | 11,447,900 (7.81) | 58,436,576 (39.88) | 14,008,438 (9.56) | 4,310,941 (2.94) | 39,346,267 (26.85) | 18,971,062  (12.95) |  |
| No. of participants in sample | 1036 | 6188 | 1610 | 596 | 7001 | 3665 |  |
| Age, years (SE) | 41.89 (0.63) | 49.88 (0.33) | 54.21 (0.45) | 32.51 (0.72) | 43.97 (0.49) | 51.92 (0.40) | <0.001 |
| Women, n (weighted %) | 661 (66.22) | 3005 (49.11) | 636 (42.12) | 349 (57.06) | 3713 (52.73) | 1890 (53.65) | <0.001 |
| Race and ethnicity, n (weighted %) |  |  |  |  |  |  | <0.001 |
| Mexican | 70 (2.91) | 533 (4.01) | 157 (4.17) | 105 (11.54) | 1456 (14.36) | 542 (10.46) |  |
| White | 608 (82.02) | 3397 (80.09) | 853 (80.03) | 239 (60.08) | 2473 (54.49) | 1528 (60.12) |  |
| Black | 68 (2.49) | 967 (6.11) | 357 (8.19) | 92 (9.96) | 1509 (14.59) | 1007 (17.07) |  |
| Other | 290 (12.58) | 1291 (9.79) | 243 (7.61) | 160 (18.42) | 1563 (16.56) | 588 (12.35) |  |
| Medical history, n (weighted %) |  |  |  |  |  |  |  |
| CVD history | 29 (2.70) | 452 (5.82) | 197 (11.22) | 15 (1.79) | 679 (7.97) | 772 (18.64) | <0.001 |
| Cancer history | 65 (7.63) | 713 (11.89) | 205 (13.41) | 26 (5.03) | 587 (8.48) | 376 (10.45) | <0.001 |
| SDoH, n (weighted %) |  |  |  |  |  |  |  |
| Unemployed | 193 (17.97) | 1525 (21.88) | 459 (26.78) | 140 (19.86) | 3129 (39.65) | 2268 (56.63) | <0.001 |
| Family income-to-poverty ratio<300% | 178 (14.14) | 1552 (18.88) | 512 (26.03) | 510 (82.08) | 6361 (87.47) | 3422 (90.74) | <0.001 |
| Marginal or lower food security | 33 (2.90) | 337 (4.35) | 143 (7.42) | 240 (36.08) | 3441 (46.43) | 2038 (53.49) | <0.001 |
| Not owning a home | 191 (16.39) | 977 (14.05) | 244 (10.27) | 379 (71.55) | 3925 (56.50) | 1988 (52.20) | <0.001 |
| Less than high school | 14 (0.89) | 259 (3.04) | 128 (7.02) | 90 (10.53) | 2348 (26.31) | 1524 (34.92) | <0.001 |
| No regular health care access | 96 (9.11) | 335 (5.89) | 82 (6.58) | 223 (37.06) | 2087 (32.65) | 785 (24.13) | <0.001 |
| No private health insurance | 90 (7.69) | 746 (10.54) | 221 (12.82) | 336 (51.61) | 5091 (69.19) | 2799 (74.00) | <0.001 |
| Not married or living with a partner | 232 (20.26) | 1192 (28.69) | 265 (16.20) | 396 (70.29) | 3850 (57.88) | 2003 (54.76) | <0.001 |
| AHA LE8 score, mean (SE) |  |  |  |  |  |  |  |
| Total CVH score | 85.21 (0.13) | 64.56 (0.18) | 42.01 (0.21) | 85.01 (0.21) | 62.73 (0.18) | 40.68 (0.22) | <0.001 |
| HEI-2015 diet score | 49.29 (0.71) | 40.66 (0.28) | 34.25 (0.52) | 46.12 (0.86) | 37.33 (0.28) | 33.07 (0.34) | <0.001 |
| Physical activity score | 95.32 (0.51) | 54.57 (1.02) | 12.44 (1.00) | 93.54 (0.73) | 43.91 (0.94) | 10.95 (0.59) | <0.001 |
| Tobacco/nicotine exposure score | 95.92 (0.38) | 81.72 (0.49) | 54.53 (1.43) | 95.28 (0.69) | 69.41 (0.94) | 41.63 (0.87) | <0.001 |
| Sleep health score | 95.34 (0.46) | 74.75 (1.48) | 49.34 (2.34) | 92.26 (0.83) | 72.09 (0.84) | 46.03 (1.61) | <0.001 |
| Body mass index score | 90.23 (0.68) | 61.50 (0.56) | 36.02 (0.99) | 91.55 (0.84) | 64.63 (0.57) | 40.47 (0.74) | <0.001 |
| Blood lipid score | 87.35 (0.78) | 62.72 (0.51) | 42.64 (0.96) | 90.75 (0.93) | 70.39 (0.52) | 45.89 (0.80) | <0.001 |
| Blood glucose score | 96.21 (0.48) | 81.78 (0.43) | 63.41 (0.93) | 97.11 (0.59) | 83.34 (0.38) | 63.20 (0.50) | <0.001 |
| Blood pressure score | 72.04 (0.34) | 58.74 (0.37) | 43.41 (0.84) | 72.43 (0.47) | 60.73 (0.35) | 44.10 (0.59) | <0.001 |

Data are survey-weighted mean (SE) or N (weight percentage %). Abbreviations: SE: standard error; SDoH: social determinants of health; CVH: cardiovascular health; AHA: American Heart Association; LE8: Life’s Essential 8; HEI-2015: healthy eating index-2015.
