# Supplementary material for: Associations of cardiovascular health and social determinants of health with the risks of all-cause and cause-specific mortality
Source: PLoS One. 2025 Nov 24;20(11):e0337286. doi: 10.1371/journal.pone.0337286 (PMC12643303; doi:10.1371/journal.pone.0337286)
Supplement: S7 Table — (DOCX) [file pone.0337286.s010.docx]

**S7 Table. Combined association of social determinants of health and cardiovascular health with all-cause and cause-specific mortality among US adults: subgroup analysis.**

| **Subgroups** | **All-cause mortality** | **CVD mortality** | **Cancer mortality** |
| --- | --- | --- | --- |
|  | **HR (95% CI)** | **HR (95% CI)** | **HR (95% CI)** |
| **Sex** |  |  |  |
| Women | 4.77 (2.32-9.81) | 8.36 (1.98-35.40) | 6.87 (1.57-30.00) |
| Men | 3.72 (2.03-6.83) | 2.73 (0.91-8.21) | 2.38 (0.83-6.81) |
| *P* for interaction | 0.691 | 0.246 | 0.413 |
| **Race/ethnicity** |  |  |  |
| White | 4.63 (2.83-7.57) | 4.00 (1.46-10.96) | 4.63 (1.70-12.57) |
| Non-white | 3.88 (1.43-10.54) | 10.33 (1.27-84.00) | 1.53 (0.37-6.25) |
| *P* for interaction | 0.916 | 0.378 | 0.383 |
| **Age** |  |  |  |
| ≥65 years | 3.19 (2.02-5.04) | 2.98 (1.31-6.78) | 3.03 (1.05-8.78) |
| <65 years | 7.70 (3.14-18.88) | 7.40 (0.99-55.20) | 7.65 (1.74-33.61) |
| *P* for interaction | 0.051 | 0.313 | 0.188 |

HR (95% CI) and *P* for interaction comparing participants with a low burden of unfavorable SDoH and high CVH vs. those with a high burden of unfavorable SDoH and low CVH are shown. Multivariate models were adjusted for age, sex, race/ethnicity, cardiovascular disease history, and cancer history. Abbreviations: SDoH: social determinants of health; CVH: cardiovascular health; HR: hazard ratio; CI: confidence interval; CVD: cardiovascular diseases.
