# Supplementary material for: Associations of cardiovascular health and social determinants of health with the risks of all-cause and cause-specific mortality
Source: PLoS One. 2025 Nov 24;20(11):e0337286. doi: 10.1371/journal.pone.0337286 (PMC12643303; doi:10.1371/journal.pone.0337286)
Supplement: S8 Table — (DOCX) [file pone.0337286.s011.docx]

**S8 Table. Combined associations of social determinants of health and cardiovascular health with all-cause and cause-specific mortality among US adults after excluding participants died within 2 years of follow-up: sensitivity analysis.**

| **Outcomes** | **CVH** | **Death/No.** | **Weighted death (%)** | **HR (95% CI)** |
| --- | --- | --- | --- | --- |
| **All-cause mortality** |  |  |  |  |
| High burden of unfavorable SDoH | High | 11/951 | 113,369 (1.09) | 1 (Reference) |
|  | Moderate | 184/5140 | 1,898,528 (3.55) | 1.53 (0.89-2.63) |
|  | Low | 78/1251 | 1,042,829 (8.12) | 2.67 (1.49-4.80) |
| Low burden of unfavorable SDoH | High | 9/558 | 78,272 (2.05) | 2.16 (0.97-4.77) |
|  | Moderate | 315/5879 | 2,157,901 (6.12) | 3.25 (1.95-5.43) |
|  | Low | 291/2672 | 2,198,530 (13.14) | 5.10 (2.98-8.73) |
| **CVD mortality** |  |  |  |  |
| High burden of unfavorable SDoH | High | 4/951 | 33,494 (0.32) | 1 (Reference) |
|  | Moderate | 45/5140 | 510,594 (0.95) | 1.26 (0.49-3.20) |
|  | Low | 20/1251 | 337,457 (2.63) | 2.54 (1.00-6.44) |
| Low burden of unfavorable SDoH | High | 0/558 | 3,764 (0.10) | 0.32 (0.04-2.75) |
|  | Moderate | 82/5879 | 751,344 (2.13) | 3.14 (1.33-7.41) |
|  | Low | 80/2672 | 611,610 (3.66) | 3.86 (1.59-9.36) |
| **Cancer mortality** |  |  |  |  |
| High burden of unfavorable SDoH | High | 2/951 | 29,304 (0.28) | 1 (Reference) |
|  | Moderate | 63/5140 | 685,719 (1.28) | 2.14 (0.76-6.00) |
|  | Low | 22/1251 | 244,020 (1.90) | 2.50 (0.83-7.48) |
| Low burden of unfavorable SDoH | High | 4/558 | 37,392 (0.98) | 3.97 (1.08-14.70) |
|  | Moderate | 77/5879 | 434,814 (1.23) | 2.65 (0.92-7.63) |
|  | Low | 62/2672 | 497,380 (2.97) | 4.94 (1.68-14.50) |

Multivariable models were adjusted for age, sex, race/ethnicity, cardiovascular disease history, and cancer history.

Abbreviations: SDoH: social determinants of health; CVH: cardiovascular health; HR: hazard ratio; CI: confidence interval; CVD: cardiovascular diseases.
