# Supplementary material for: Associations of cardiovascular health and social determinants of health with the risks of all-cause and cause-specific mortality
Source: PLoS One. 2025 Nov 24;20(11):e0337286. doi: 10.1371/journal.pone.0337286 (PMC12643303; doi:10.1371/journal.pone.0337286)
Supplement: S9 Table — (DOCX) [file pone.0337286.s012.docx]

**S9 Table. Combined associations of social determinants of health and cardiovascular health with all-cause and cause-specific mortality among US adults after excluding participants with cardiovascular diseases or cancer: sensitivity analysis.**

| **Outcomes** | **CVH** | **Death/No.** | **Weighted death (%)** | **HR (95% CI)** |
| --- | --- | --- | --- | --- |
| **All-cause mortality** |  |  |  |  |
| High burden of unfavorable SDoH | High | 19/954 | 97,928 (0.95) | 1 (Reference) |
|  | Moderate | 307/5714 | 1,253,886 (2.55) | 1.35 (0.66-2.76) |
|  | Low | 144/1494 | 563,178 (5.18) | 2.05(1.09-4.71) |
| Low burden of unfavorable SDoH | High | 14/544 | 47,701 (1.18) | 2.15 (0.73-6.36) |
|  | Moderate | 484/6331 | 1,415,581 (4.21) | 3.21 (1.55-6.67) |
|  | Low | 485/3289 | 1,400,216 (9.90) | 5.26 (2.53-10.90) |
| **CVD mortality** |  |  |  |  |
| High burden of unfavorable SDoH | High | 6/954 | 32,987 (0.32) | 1 (Reference) |
|  | Moderate | 94/5714 | 239,235 (0.49) | 0.64 (0.17-2.49) |
|  | Low | 46/1494 | 167,836 (1.54) | 1.45 (0.33-6.33) |
| Low burden of unfavorable SDoH | High | 1/544 | 0 | 0 |
|  | Moderate | 156/6331 | 362,713 (1.08) | 1.94 (0.49-7.58) |
|  | Low | 144/3289 | 390,495 (2.76) | 3.42 (1.07-13.40) |
| **Cancer mortality** |  |  |  |  |
| High burden of unfavorable SDoH | High | 5/954 | 15,223 (0.15) | 1 (Reference) |
|  | Moderate | 95/5714 | 451,473 (0.92) | 3.01 (0.56-16.10) |
|  | Low | 34/1494 | 164,338 (1.51) | 3.68 (0.67-20.20) |
| Low burden of unfavorable SDoH | High | 5/544 | 24,793 (0.62) | 7.19 (0.92-56.10) |
|  | Moderate | 106/6331 | 337,724 (1.00) | 4.50 (0.86-23.70) |
|  | Low | 111/3289 | 277,661 (1.96) | 6.09 (1.15-32.10) |

Multivariable models were adjusted for age, sex, race/ethnicity.

Abbreviations: SDoH: social determinants of health; CVH: cardiovascular health; HR: hazard ratio; CI: confidence interval; CVD: cardiovascular diseases.
