# Supplementary material for: Associations of cardiovascular health and social determinants of health with the risks of all-cause and cause-specific mortality
Source: PLoS One. 2025 Nov 24;20(11):e0337286. doi: 10.1371/journal.pone.0337286 (PMC12643303; doi:10.1371/journal.pone.0337286)
Supplement: S10 Table — (DOCX) [file pone.0337286.s013.docx]

**S10 Table. Combined associations of social determinants of health and cardiovascular health with all-cause and cause-specific mortality among US adults adjusted for more covariates: sensitivity analysis.**

| **Outcomes** | **CVH** | **Death/No.** | **Weighted death (%)** | **HR (95% CI)** |
| --- | --- | --- | --- | --- |
| **All-cause mortality** |  |  |  |  |
| High burden of unfavorable SDoH | High | 22/1036 | 159,137 (1.39) | 1 (Reference) |
|  | Moderate | 352/6188 | 2,262,059 (3.87) | 1.32 (0.84-2.09) |
|  | Low | 169/1610 | 1,233,621 (8.81) | 2.24 (1.34-3.75) |
| Low burden of unfavorable SDoH | High | 18/596 | 97,362 (2.26) | 1.95 (0.98-3.89) |
|  | Moderate | 608/7001 | 2,771,029 (7.04) | 2.92 (1.83-4.65) |
|  | Low | 594/3665 | 2,6292,749 (14.19) | 4.31 (2.70-6.86) |
| **CVD mortality** |  |  |  |  |
| High burden of unfavorable SDoH | High | 7/1036 | 53,379 (0.47) | 1 (Reference) |
|  | Moderate | 99/6188 | 528,653 (0.90) | 0.81 (0.31-2.11) |
|  | Low | 52/1610 | 401,616 (2.87) | 1.82 (0.69-4.85) |
| Low burden of unfavorable SDoH | High | 1/596 | 3,764 (0.09) | 0.20 (0.02-1.79) |
|  | Moderate | 188/7001 | 871,876 (2.22) | 2.16 (0.87-5.41) |
|  | Low | 171/3665 | 708,623 (3.74) | 2.62 (1.04-6.56) |
| **Cancer mortality** |  |  |  |  |
| High burden of unfavorable SDoH | High | 5/1036 | 29,304 (0.26) | 1 (Reference) |
|  | Moderate | 121/6188 | 833,824 (1.43) | 2.64 (0.95-7.32) |
|  | Low | 44/1610 | 317,123 (2.26) | 3.27 (1.12-9.53) |
| Low burden of unfavorable SDoH | High | 6/596 | 39,768 (0.92) | 4.36 (1.22-15.60) |
|  | Moderate | 138/7001 | 613,445 (1.56) | 3.78 (1.33-10.70) |
|  | Low | 137/3665 | 594,927 (3.14) | 5.96 (2.09-17.00) |

Multivariable models were adjusted for age, sex, race/ethnicity, cardiovascular disease history, cancer history, drinking status, chronic kidney disease (CKD), chronic obstructive pulmonary disease (COPD). Drinking status was classiﬁed as current drinkers or noncurrent-drinkers. CKD was defined as meeting one of the following: estimated glomerular filtration rate (eGFR) < 60 mL/min/1.73 m^2^ or urinary albumin-to-creatinine ratio (ACR) ≥ 30 mg/g. COPD was self-reported history of COPD.

Abbreviations: SDoH: social determinants of health; CVH: cardiovascular health; HR: hazard ratio; CI: confidence interval; CVD: cardiovascular diseases.
